# Experiences of diagnosis and treatment for upper limb Complex Regional Pain Syndrome: a qualitative analysis

**DOI:** 10.1093/pm/pnad111

**Published:** 2023-08-16

**Authors:** Grace S Griffiths, Bronwyn L Thompson, Deborah L Snell, Jennifer A Dunn

**Affiliations:** Department of Orthopaedic Surgery and Musculoskeletal Medicine, University of Otago, Christchurch 8011, New Zealand; Department of Orthopaedic Surgery and Musculoskeletal Medicine, University of Otago, Christchurch 8011, New Zealand; Department of Orthopaedic Surgery and Musculoskeletal Medicine, University of Otago, Christchurch 8011, New Zealand; Department of Orthopaedic Surgery and Musculoskeletal Medicine, University of Otago, Christchurch 8011, New Zealand

**Keywords:** Complex Regional Pain Syndrome, upper limb, healthcare experience, hand therapy, rehabilitation

## Abstract

**Introduction:**

Complex Regional Pain Syndrome (CRPS) most frequently affects the upper limb, with high associated disability. Delays to diagnosis and appropriate treatment can adversely impact prognosis and quality of life, but little is known about the healthcare experiences of people with CRPS. This study aimed to explore lived experiences of diagnosis and treatment for people with upper limb CRPS.

**Methods:**

Participants were recruited through online support groups and multiple public and private healthcare settings in the Greater Wellington Region, New Zealand. Semi-structured interviews were conducted with participants who had experienced upper limb CRPS for more than three months and less than three years. Interviews were transcribed verbatim and analysed using reflexive thematic analysis.

**Results:**

Thirteen participants (11 female, 2 male) aged between 43 and 68 years were interviewed. Duration of CRPS ranged from 7 months to 2.5 years. Five themes were identified. Participants initially engaged in healthcare out of a desire to return to being the person they were before having CRPS. Three interacting experiences epitomised the overall healthcare experience: (1) not knowing what is going on, (2) not being taken seriously, and (3) healthcare as adding another layer of load. Meanwhile, participants used multiple approaches in an attempt to not let CRPS stop them from continuing to live their lives.

**Conclusions:**

Participants in this study felt that credible information, validation, and simplification from healthcare providers and systems would support their process of navigating towards a meaningful life and self-concept in the presence of CRPS.

## Introduction

Complex Regional Pain Syndrome (CRPS) is a pain condition characterised by severe pain and sensory, motor, and autonomic changes.^1^ It occurs most frequently following limb injury, although the trauma can be mild, and sometimes without initiating event.^2^ Risk factors include, but are not limited to, early uncontrolled baseline pain, crush injuries, severe fractures, immobilisation, and female gender.^3^^,^^4^ CRPS is thought to involve an interplay between aberrant inflammatory mechanisms, vasomotor dysfunction, and maladaptive neuroplasticity.^5^ Due in part to this complexity, CRPS can be difficult to diagnose and treat.^6^ The diagnostic criteria widely recognised today (the Budapest/New International Association for the Study of Pain [IASP] criteria) was proposed in 2003 in an attempt to standardise diagnosis.^7^^,^^8^ However, there remain delays to recognition, and, in turn, commencement of appropriate treatment.^9^^,^^10^ Such delays can lead to poorer outcomes and medicolegal complications; thus, early recognition and intervention is critical to reducing long-term disability and psychosocial distress.^11^ CRPS is associated with high health costs and work incapacity,^12^ severe pain, significant disability,^13^ and high risk of suicide.^14^ It most frequently affects the upper limb,^15^ disability of which has profound consequences for activities of daily living and wellbeing.^16^ Thus, CRPS of the upper limb may be particularly debilitating. Studies exploring lived experiences of CRPS often feature challenges to receiving a diagnosis and appropriate treatment.^13^^,^^17–19^ Calls have been made for more lived experience research to enhance understanding and enable development of appropriate national pain strategies and treatment guidelines.^20^ Thus, the aim of this study is to explore lived experiences of diagnosis and treatment for people with upper limb CRPS in New Zealand.

## Methods

### Study design

This qualitative study formed the first phase of an exploratory mixed methods design, underpinned by a critical realist methodology. Ethical approval was gained through the University of Otago Human Research Ethics Committee (H21/177).

### Researcher declaration

The authors hold dual roles as clinician-researchers,^21^ with professional backgrounds in occupational therapy (G.G. and B.L.T.), physiotherapy (J.D.) and psychology (D.S.), including specialisation in upper limb rehabilitation (G.G. and J.D.) and pain management (B.L.T.). To promote study rigour, we met regularly during study design and implementation to reflect upon our interests, beliefs, and assumptions and thus make explicit the ways in which these influence the research process.

### Participants

Participants were recruited between March and September 2022 from the Greater Wellington Region, New Zealand. Multiple strategies were used to maximise response rate, including emailing study information to relevant boards, societies, and online support groups, and in-person visits to all hand therapy, physiotherapy, pain management, and specialist services in the region. Healthcare providers were asked to share study information with potential participants, and, with consent, to share their contact details with the research team. Participants were considered eligible if they were 18 years or older; had a confirmed diagnosis of CRPS (as per the Budapest/New IASP criteria) of the upper limb (defined as fingertip to shoulder/axilla); had been diagnosed for more than 3 months (to ensure a range of experience) and less than 3 years (to limit recall bias); and had recently received hand therapy, physiotherapy, psychology, and/or occupational therapy input.

### Data collection

Participants provided written informed consent and attended one semi-structured interview with GG at a mutually convenient location (see Supplemental Appendix A). Interviews were audio-recorded and then transcribed verbatim by GG and returned to participants for member-checking. To maintain anonymity participants were allocated a pseudonym reflecting their self-identified gender. Interviewing continued until no new codes were identified; thus ensuring data saturation.^22^

### Data analysis

**Figure 1.**
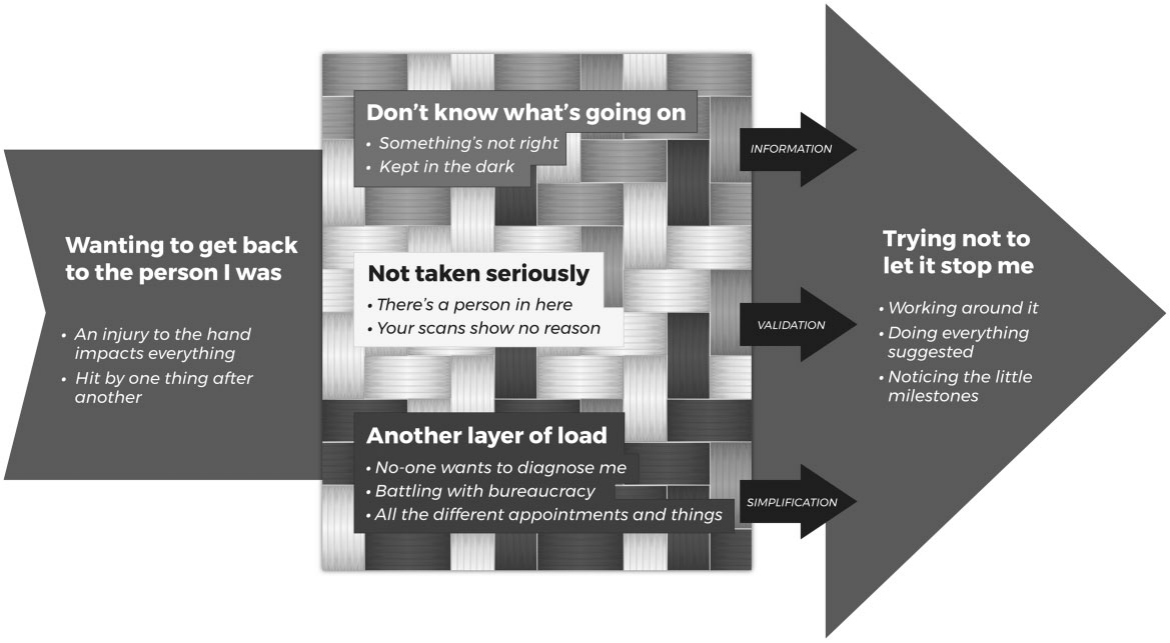
Proposed model of the lived experiences of diagnosis and treatment for upper limb Complex Regional Pain Syndrome. The centre woven rectangle represents the interaction between interview participants’ three overall healthcare experiences. The smaller arrows correlate to each healthcare experience and reflect what interview participants most desired to support them in their CRPS journey. The large arrow in the background represents participants’ initial and ongoing process of navigating towards a meaningful life and self-concept in the presence of CRPS.

We employed a 6-stage reflexive thematic analysis of interview transcriptions: data familiarization, coding, generating initial themes, reviewing themes, defining and naming themes, and writing up.^23^ First G.G. and J.D. read and reread the entire interview data set, writing notes of initial thoughts, and discussing potential findings. Then, using NVivo software, G.G. coded the entire data set by identifying broad categories in the data and labelling data portions within these with in-vivo codes. J.D. independently coded 25% of the interviews. G.G. and J.D. regularly met to check consistency across coding and discuss developing themes. The research team then met to discuss, identify, and log prototype themes and supporting quotations (see Supplemental Appendix B). We then developed a figure to illustrate how themes interact (Figure 1), which interview participants reviewed to ensure that their meanings were accurately captured.

## Results

### Participant characteristics

Thirteen people (11 women, 2 men) were interviewed. Interviews lasted between 45 and 60 minutes. Mean age of participants was 55 years (range 39–68 years), and mean time since CRPS onset was 19 months (range 7–26 months). Participant characteristics are summarized in Table 1.

**Table 1.** Characteristics of participants.

Characteristic	*n* (%) or mean (SD) *n* = 13
Mean age at interview, years [(SD), range]	55 (8.8), 39–68
Median age at interview, years	56.5
Gender (n [%])	
−Female	11 (85%)
−Male	2 (15%)
Ethnicity (n [%])	
−NZ European	10 (72%)
−Māori	1 (8%)
−Pasifika	1 (8%)
−European	1 (8%)
−Unknown	1 (8%)
Initial injury	
−Fracture (wrist)	5 (38%)
−Fracture (elbow/humerus)	2 (15%)
−Sprain or crush (finger/wrist)	5 (38%)
−Elective surgery	1 (8%)
Mean time since injury at date of interview, months [(SD), range]	19 (7.5), 7–26

### Interview results

The theme “Wanting to get back to the person I was” explained participants’ initial reasons for engaging with healthcare, with subthemes titled “An injury to the hand impacts everything” and “Hit by one thing after another”. Three interacting themes then described participants’ experiences of diagnosis and treatment. These were first “Don’t know what’s going on,” with subthemes titled “Something’s not right” and “Kept in the dark”; second “Not taken seriously,” with subthemes titled “There’s a person in here” and “Your scans show no reason”; and third, “Another layer of load,” with subthemes titled “No-one wants to diagnose me,” “Battling with bureaucracy” and “All the different appointments and things”. The final theme “Trying not to let it stop me,” with subthemes titled “Working around it,” “Doing everything suggested,” and “Noticing the little milestones,” describes participants’ continuous navigation process, influenced by their experiences of healthcare (Figure 1). Supporting data for each theme and subtheme are shown in Table 2.

**Table 2.** Themes, sub-themes, and supporting data.

Theme	Subtheme	Supporting data
Wanting to get back to the person I was	An injury to the hand impacts everything	I don't like being at home. Without a job … you've got to have money and you've got to have your self-worth, and work is self-worth for a lot of people, and it is for me. Being at work means, for me, that I'm around other people. At home I’m on my own. So, it’s quite the social thing really, being at work, you know, working. So there was the loss of that as well, with the injury. (Kimberly) I’m a very independent, self-sufficient individual. I’ve been on my own for a while now. And I’m self-sufficient, I can do DIY around the house, I can do, I can fix things, I can do—and I couldn't do things? And I had to have people help me? … But yeah, the autonomy, the self-sufficient side of life, has really cut back. (Joan) But, because it was in my right hand, and I’m right-handed, my ability to do the hobbies and the things that I love, all of a sudden came to a screaming halt. (Rosemary)
	Hit by one thing after another	I've had other things crop up. And they now believe that the stress of it all, being in pain for so long, and having to deal with the pain for so long, the lack of sleep and everything, and it’s just caused the brain to a massive hissy fit. (Tania) Things that I used to rely on for my left hand, my right hand’s had to pick up on. And because of that my right hand has ended up with carpal tunnel as well. So it’s kind of like, I'm going from one thing, bouncing to another all the time. And it’s like, is there light at the end of the tunnel? (Kimberly)
Don’t know what’s going on	Something’s not right	When I got the cast off … it looked so bad … it actually looked dead. It looked so awful. And [the doctor] sent me away from the hospital—like he gave me a sling, but … there was no brace, there was nothing on it. I felt sooo vulnerable? …. I turned up at work straight after that, coz I had to get back there, and they looked at it, and they were like—[pause, shocked expression] that doesn’t look right. And I was like, I know it doesn’t look right, I know it doesn’t look right! And they were like why didn’t they give you a brace or something? I was like I don’t know! (Stacey) …by week four I had pretty much lost all movement in the wrist and the fingers and the thumb, and everything had just gone stiff and swollen …. And very, very - extremely- I have never had pain like it before …. I was wondering what the hell was going on. (Tania)
	Kept in the dark	And then he said to me, oh your doctor would have explained all this. And my ears sort of went—doctor? Explained? What? You know, I guess I was a wee bit slow on the uptake, but of course I just wasn't really clear on it all. And I thought, no, the doctor hasn't explained anything to me. So then I came home and ruminated and ruminated, so then I rang the doctor surgery, and I was a bit annoyed then, because I had been having this feeling that I'd been in, and that I was in the dark a wee bit? (Angela)
Not taken seriously	There’s a person in here	And the nurse who was cutting [the cast] off wasn’t like the other nurses, and I think this is really important, that the other nurses were over gentle, like way more gentle than they needed to be. And she wasn’t? … I said to her, please don’t angle the scissors that cut the cast. My skin is hypersensitive …. And I’m really scared that you’re gonna, it feels like you’re ripping—gonna rip the skin. And she wasn’t very happy with me. (Megan) I just think that … they need to be more aware of what they say to their patients? And how they say it, so that we do have more understanding, instead of them coming across a lot of the time as being neglectful, arrogant, or just disinterested in what’s going on with the people, we’re just another number. (Kimberly) I think I, I feel like I was dismissed as a bit of a neurotic, middle-aged person. And that’s super unfair. Because they do not know, they don’t know you. And I just think that being able to, and I know that people probably get a bit numb to it, but it’s a really important part of the job to listen. (Stacey)
	Your scans show no reason	They even sent me for an MRI scan. And that came back with nothing. And that’s when they turned around and like, not in so many words, but it was kind of like it was all in my, all in my head. Sometimes I felt like just walking out of the physio when you hear things like that. (Richard) Just the way that I was treated by X [hand therapy clinic], by doctors, by surgeons as well. Basically they would look at MRI results, they would look at the x-rays, and they would say, well, you know, everything seems to be healing. (Kimberly)
Another layer of load	No-one wants to diagnose me	She didn’t talk to me about CRPS until she had suspected for 2–3 weeks … it must be possible to have identified me as a potential. Even if you’re like well I think that [Megan] is probably a 70% candidate … (Megan)
	Battling with bureaucracy	And I had a [funder] specialist review …. He started playing games and semantics ….They actually sent me a letter saying we’re going to decline cover because we’re outside of the [timeframes] …. So I ended up losing it and saying you need to stop. You need to stop playing games. I’m a real person at the end of this. I’m in a lot of pain. Act with integrity. (Roger) So now I've got to try and work out how I get my income, what it should have been, through [funder]. So now I've got to do a battle with [funder], no doubt, in my income. So I tried to ask some questions, and … they're not exactly helpful. So I think [funder] need to be able to come to the party and go, look, here's your claim, and because it's a [specific type of claim], here is a booklet of what your entitlements are. And it should be automatic. (Tania)
	All the different appointments and things	I was under the [funder] team. Normally it would take up to an hour for them to answer the phone … it was always a different person, and all promises would be made, nothing would happen. Then I would repeat, and repeat, and repeat. (Roger) But it’s hard to remember what they've actually said, because you have so many conversations with so many different people, you forget what one person from the next says. (Richard)
Trying not to let it stop me	Working around it	I’ve decided to take the view that I can, like, kind of work through this? And just take one step at a time and not get too freaked out? But mainly the information has been really powerful for me, in terms of just seeing more progress once I got my head around, like, the nature of what it is. So I could then ask my body to do things and go I know this actually does not feel good, but it will be okay, and this is the way to get better? Yeah. Like, just starting to participate more fully in things and not shy away from it coz I have an injury? (Stacey) Hanging washing out. Just a nightmare. And, but I did it, and then I would sit there for the next 2 days not being able to move my hand coz it was all so swollen. And then I worked out other ways of doing things. Put things on hangers, instead of hanging them up using pegs. (Joan)
	Doing everything suggested	I’ve had to teach my hand about hot things and cold things. Because everything was to the absolute extreme. You touch something cool, and it would feel frozen. Something slightly warm would feel boiling hot. Get into the shower. Touch everything with my right hand. This is normal. Have to teach the [left] hand that this was, this is normal …. So the temperature changes aren’t so extreme now …. I would say the massage machine was most effective, because I was in control of that. I mean, just even touching the skin was absolutely agony. Um, but bit by bit that really improved. (Tania) My sister-in-law told me, you've got a keyboard, use it to do exercises for your finger. But I go but it hurts. And she went, it's good exercise for you. But now I've really been enjoying playing the keyboard, which is quite good. (Eseta)
	Noticing the little milestones	The day I carried my coffee cup, the first time when I actually carried it, carrying my coffee cup about 5 metres, and I hadn’t, and then I stopped and went that [added left hand to support weight]. But the day that I carried my cup of tea, I went [big smile]. (Joan)

#### Wanting to get back to the person I was

All participants initially engaged with healthcare out of a desire to return to being the person they were before CRPS. This sense of self was expressed through previous occupations, roles, and ways of doing: “I wanted to get back to being the person I was before the injury…. getting back to work, being able to go out in my garden and not pace myself” (Joan). Participants reported their sense of self was primarily altered by the loss of upper limb function, and the development of new health complexities. These are described further in the subthemes: an injury to the hand impacts everything, and hit by one thing after another.

- *An injury to the hand impacts everything.* Participants described the life-changing impact of lost upper limb function on their usual occupations and roles: “When I had the injury, as you can imagine, an injury to a hand impacts everything. You can’t even bloody button up your trousers” (Megan). Changes to activity performance impacted participants’ sense of self; Eseta described playing her guitar as “how I breathe,” ascribing a spiritual significance to this activity and expressing profound grief at its loss.
- *Hit by one thing after another.* Many participants described CRPS as a catalyst for other health issues, which added complexity to their lives and further eroded their sense of self. These included developing pain in the other upper limb (Joan, Kimberly, Liz) and previous surgery sites (Roger), and neurological issues: “My doctor thinks the brain’s just going, nah, we’ve had enough…. we’re just getting hit with one thing after another” (Tania). These participants were uncertain whether these health concerns were “related to the CRPS” (Angela), “side effects of the pain medication” (Liz), prolonged sleep deprivation, or other causes.

#### Don’t know what’s going on

All participants described their CRPS journey being characterised by unexpected challenges and unanswered questions. Information appeared to influence whether participants felt their concerns were taken seriously by healthcare professionals, and whether healthcare was perceived as helpful or not. Most participants initially assumed, or tried to convince themselves, that their experience was normal, feeling this was due to a lack of information about what might signal an unusual recovery path.

I didn’t know why I was having excessive sweating … and I assumed that the swelling was what comes after you’ve had the broken bones …. But, you don’t know what's going on coz you’re sort of a bit unclear yourself, and the pain is, you know, quite a dominant feature. (Angela)

Participants expressed an ongoing desire for information once becoming aware that something was wrong. This is described further in the subthemes: something’s not right, and kept in the dark.

- *Something’s not right.* Participants described becoming aware of unexpected symptoms, including uncontrollable pain, sensitivity, swelling and/or pressure from a cast, visual and temperature changes to the limb, and the limb feeling foreign, stiff, shaky, and/or difficult to move. “I knew something wasn’t right, the cast was really tight the whole way through … I didn't know what was going on with my body…” (Megan). Several participants described their need for information at this point being heightened by co-occurring challenges with sleep, work or household task performance, and financial/medicolegal concerns.
- *Kept in the dark.* Participants expressed feeling “powerless” (Stacey) and lacking “control” (Liz) over their limb and its progress, while sometimes sensing an imbalance between themselves and healthcare providers: “I just felt like everybody else knew but they wanted to keep me in the dark like a mushroom” (Kimberly). Two participants (Robyn, Tania) described finding out about their condition “in an underhand way” (Tania), by reading communication between healthcare providers. All participants wanted more information, to enable involvement in their own healthcare. Participants especially desired information regarding the nature and prognosis of CRPS, as this was perceived as influencing how they chose to manage it: “if it’s something I just need to learn how to deal with, then … I need to, either with their [healthcare providers’] help or without it, figure it out…” (Robyn).

#### Not taken seriously

Many participants described interactions with healthcare providers that left them feeling invalidated: “you just think to yourself what's the point of going on here because nobody seems to take you seriously” (Richard). These experiences influenced whether information provided was perceived as helpful. Two subthemes exemplified this: there’s a person in here, and your scans show no reason.

- *There’s a person in here.* Participants described interactions where they felt healthcare providers were doing or saying things without considering the impact or listening to the concerns of the person.
  When they realigned the bone … there was an awful nurse there who … was very busty, and no nonsense. … probably coz she sees so many people with broken bones, she knows what she's got to do, but she’s forgotten it’s a body and a person in there? (Joyce)
  Dismissive interactions led to participants interpreting explanations about their symptoms as “all in your head” (Kimberly, Richard). Conversely, healthcare providers taking the time to have honest conversations with participants showed that they were being heard, their condition was being acknowledged as real, and the personal impact was understood. Such validation was explicitly described by participants as being the most helpful aspect of their healthcare experience (Liz, Joyce, Stacey).
  I sat there and spoke to him and told him everything. And we worked out what I could and couldn't do. And he never once mentioned anything to with it’s all in my mind or anything, he was actually quite nice, he was talking to me like a proper human being …. (Richard)
- *Your scans show no reason*. Participants often perceived “being told basically you’ve got a malfunction in your brain” (Kimberly) when they failed to fit expected healing timeframes, and in the absence of “something concrete” (Robyn) identified on imaging results. This experience was described most strongly by participants who did not feel heard, understood or validated: “He said, your x-ray, your scans, show no reason for you to not be able to play your guitar. And I went, oh my gosh, there's something wrong with me, something wrong with my head” (Eseta). Robyn suggested that healthcare providers further consider how to discuss inconclusive imaging results: “…in a way that doesn't start to make you question whether or not you’re sane.”

#### Another layer of load

Participants described navigating healthcare systems and processes on top of struggling with symptoms associated with CRPS as “another thing to look after … another layer of load” (Stacey). Three subthemes illustrated this: no-one wants to diagnose me, battling with bureaucracy, and all the different appointments and things.

- *No-one wants to diagnose me.* Many participants described sensing reluctance from healthcare providers to discuss CRPS even if they were suspecting and/or treating it: “I don't think anyone actually wants to diagnose you? They want someone else to do it?” (Joan). Participants who received their CRPS diagnosis relatively quickly and without significant contradiction described feeling relief (Megan, Liz, Rosemary, Joyce), explaining “when something has a name, it’s much easier to deal with than just a whole bunch of symptoms” (Liz). The two participants who had the longest delay to CRPS diagnosis responded with anger, interpreting it as a psychosomatic diagnosis (Richard, Kimberly).
- *Battling with bureaucracy.* Most participants described going back and forward between funders and healthcare providers to justify ongoing treatment for a condition that defied normal injury healing timeframes: “and the problem with [funder] is they expect me to be healed in this defined period of time. And it doesn’t work like that, not with CRPS” (Roger). Those that had to fight for acceptance of their CRPS diagnosis on their injury claim felt that this both impacted their wellbeing at the time and delayed access to treatment, ultimately worsening their prognosis: “You have to have a lot of energy to be able to fight that system” (Joyce). Once participants had their CRPS claim accepted by funders they described how this positively impacted their ability to access ongoing treatment and get on with life, without having their diagnosis and limitations constantly questioned, or needing to repeatedly complete complicated forms (Joan, Tania, Joyce).
- *All the different appointments and things.* Participants frequently articulated the additional difficulties associated with travelling to and juggling multiple appointments. These included repeating themselves to different healthcare providers, constantly building new relationships (due to high staff turnover or referrals), and assimilating conflicting information from these multiple sources: “I mean, it’s time, it’s money …. It’s a pain in the arse to have to go to all the different appointments and things …. I wouldn't be going if I didn't have to” (Robyn). Richard suggested that “just dealing with one person” would reduce the burden of having to say “the same thing time and time again” (Roger), and failing this, a “coordinated approach” (Roger) and having “everyone on board” (Joyce) would reduce the load borne by participants.

#### Trying not to let it stop me

Participants described trying to navigate towards goals representing who they were or who they wanted to be. Frequently, these goals represented valued life roles, such as parent, partner, friend, and/or employee: “I try not to let it stop me from cooking, and, you know, I still have to cook for my son and my daughter” (Eseta). Three subthemes demonstrated this process: working around it, doing everything suggested, and noticing the little milestones.

- *Working around it.* To move forward participants described finding workarounds, both naturally: “your body works around” (Stacey), and deliberately: “I had to work out ways of doing things” (Joan).
  I’m not back to normal, and the things I like doing, you know, are challenging, to say the least. But I'm kinda thinking, ok, well I'll do 10 minutes, and see how I go …. I think I've worked around it; I've found other things that make me happy. (Joyce)
  Sometimes participants resisted these adaptations, preferring to “try to carry on as normal, and ignore it” (Kimberly). These attitudes and resultant strategies adopted appeared connected to participants’ beliefs about CRPS, how many treatments or strategies they had already tried and to what effect, and their ultimate priorities for their hand.
- *Doing everything suggested.* Participants described using a range of treatments to reduce symptoms and improve movement and function: “You name it, I’ve had it …. I’ve done everything anyone’s suggested” (Joyce). Participants appeared to prefer approaches that connected to their existing values: “I’m a talker. I like narrative therapy. So, it really gelled with me” (Joyce), and described selecting approaches that resonated while “ditch[ing] things that don’t really work for me” (Stacey). Similarly, participants most frequently described therapies that they performed in their own life contexts, using ready-to-hand props, and combining more than one therapeutic medium. Angela, who described herself as “a swimmer” pre-injury, combined her movement exercises with heat in her bath at home. Megan described “the explanation of what it [CRPS] was” as being “the one key to unlock a whole lot of other stuff …. Because that gave me the understanding to be creative and come up with my own ways of dealing with it.”
- *Noticing the little milestones.* Participants frequently described measuring changes to their CRPS by their ability to grip, hold, and lift with their hand. They appeared to translate these functional movements into their own lives as they described milestones that stood out to them and the “sense of achievement” (Richard) meeting these brought.
  I’m sometimes forgetting, like I'll pick up a shopping bag or something and I’ll be like, I just did that with the bung arm and it was okay! So I will notice the little milestones, funny ones, like being able to put on my bra like, that way [arms behind back]. (Stacey)
  Participants’ rehabilitation priorities appeared to change over time, shifting from symptom reduction to increased use, even in the absence of complete symptom resolution.

## Discussion

The findings from this study indicate that people with upper limb CRPS initially engage with healthcare from a desire to return to being the person they used to be. Central to the healthcare experience is whether a person knows what is happening (in their physical body and with their healthcare). This information influences whether people feel like they are being taken seriously; a sense of validation which in turn influences how information is received and whether healthcare systems and processes are experienced as another layer of load. These experiences either contribute to or detract from the person’s own process of navigating toward being the person they want to be.

The finding that people with upper limb CRPS desire to return to being the person they used to be is consistent with studies that have separately explored the impact of CRPS,^24^ upper limb disability,^25^ chronic pain^26^ and other chronic health issues^27^ on people’s sense of self. The upper limb has long held a spiritual significance;^28^ it performs, communicates, and experiences in ways unlike any other part of the body. Thus, the combination of functional, social, and sensory issues common with upper limb CRPS has a major impact on self-concept. In response, participants in this study described steps they took to move forward despite their CRPS. At the forefront of this process was participants’ prior ways of being and doing, own preferences for rehabilitation, and own priorities for measuring progress. Alongside grief for lost self was recognition that getting back to being the person they were might look different than initially expected, and determination to reclaim valued aspects of self. This process has been described elsewhere in the chronic pain literature as an adjustment to persistent pain and limitations while renegotiating self-concept.^29^ As echoed by other studies, this process appears to be helped or hindered by healthcare experiences along the way, in particular, by validation of both pain and self,^30^ and by a person’s understanding of treatment.^24^ In this study, participants articulated three key elements that impacted their experience of diagnosis and treatment for upper limb CRPS. These were information, validation, and simplification of healthcare processes.

### Information

All participants expressed a desire to have received more information from their healthcare providers. Participants had different priorities for information at different stages of their journey. Initially, they wanted information about what to look out for to indicate an abnormal injury trajectory, to explain and monitor their symptoms. Then, they wanted information about what was causing their symptoms, what treatments were available and how to access these. Finally, they wanted to know about their prognosis. These findings are similar to those of Moore et al., where people with CRPS desired an explanation, evidence-based treatment and self-management options.^31^ However, as in the study by Johnston-Devin et al., our participants felt that healthcare providers themselves don’t know enough about CRPS or chronic pain in general, and that their resulting uncertainty effects participants’ experiences.^32^ In our study, understanding what was going on was central to the overall diagnosis and treatment experience, and facilitated participants’ active, informed involvement in their own healthcare. Conversely, participants who had a limited understanding of what was going on expressed distress, disability, and despair. Studies show that a poorer understanding of CRPS is associated with greater disability and kinesiophobia,^33^ while greater pain catastrophising predicts pain and disability in people with upper limb CRPS.^34^ If information impacts beliefs and psychological responses to CRPS, knowing what information people desire and delivering this in an effective and timely way is essential.

### Validation

In this study, being provided with information showed participants they were being taken seriously and provided validation. However, the way information was provided was equally important. Participants emphasized the impact of a consistent relationship, patience, and careful selection of words, echoing the findings of Johnston-Devin et al.^32^ These were especially important when explaining scan results in the absence of visible injury. This situation is known in the chronic pain literature to be potentially invalidating, necessitating considerable empathy.^35^ It requires interpretations to extend beyond reductionist explanations of pain, which may fuel stigma.^36^ Participants in our study agreed the attitudes and behaviours of healthcare providers were the most influential aspect of their diagnosis and treatment experiences. However, there was no consensus on which treatments were most beneficial. While algorithms have been proposed to improve the effectiveness of CRPS treatment,^37^ suggesting symptom-based or even mechanism-based treatment,^38^ findings from our study support further emphasis of person-centred treatment.^39^ Providing individualised, holistic care is within the scope of all healthcare providers, and is especially important given the emotional impact of CRPS.^40^

### Simplification

Participants in our study described how engaging with healthcare—navigating diagnosis, bureaucracy, and multiple appointments—added another layer of load. Participants expressed a desire for a simplified process. Again, this need was influenced by both information and validation. Participants felt they should have been told about CRPS and referred to appropriate services earlier, even in lieu of a formal diagnosis. However, as in other pain conditions,^41^ the journey to receiving a diagnosis and treatment was, for most, long and circuitous, requiring people to navigate multiple processes and systems. This often led to frustration and sensing prejudice, as described elsewhere,^42^ particularly where insurance or funding was involved.^43^ Given the experience of living with CRPS has been described as a battle,^13^ streamlining the process of diagnosis and receiving treatment to minimise bureaucratic battles may reduce cognitive load and a contributing sense of injustice/stigma.^44^ This requires clearer pathways, straightforward claims acceptance, and a unified team approach.^45^ In the absence of an interdisciplinary team approach, enhanced communication between providers, consistent messaging, and documenting signs and symptoms according to diagnostic criteria are recommended.

There were 3 main limitations to this study. To reduce recall bias, we deemed people who had not accessed healthcare in the past 3 months ineligible for this study. This may have unintentionally excluded the experiences of participants who had chosen not to continue with treatment. Second, our inclusion criteria intended to make the findings particularly relevant to certain rehabilitation providers who may be most likely to treat CRPS. However, this may have unintentionally excluded people who accessed alternative/cultural treatments. Finally, the experiences of people who were not referred for treatment in the first place are not represented in this study. Despite these limitations, there are many similarities between our findings and those of other studies already discussed.

## Conclusions

Upper limb CRPS significantly changes people’s lives and self-concept, leading people to engage with healthcare while embarking on their own navigation process. In this study, participants described the challenges of trying to fit themselves into the existing healthcare system, and desired a combination of information, validation, and simplification to support them in moving forward with CRPS. This indicates a need for a paradigm shift in CRPS management, where healthcare providers consciously enter individuals’ lives and fit treatment into this. These findings lead to the following recommendations: for provision of timely, holistic, and credible information about CRPS to individuals, greater emphasis on the therapeutic alliance between the individual and healthcare providers, and a unified team approach to simplify and improve healthcare for CRPS.

## Supplementary Material

pnad111_Supplementary_Data
