# Supplementary material for: Experiences of diagnosis and treatment for upper limb Complex Regional Pain Syndrome: a qualitative analysis
Source: Pain Med. 2023 Aug 16;24(12):1355–63. doi: 10.1093/pm/pnad111 (PMC10690851; doi:10.1093/pm/pnad111)
Supplement: pnad111_Supplementary_Data [file pnad111_supplementary_data.zip › pnad111_Supplementary_Data/Appendix A Interview Questions.docx]

**Appendix A: Interview questions**

**Initial injury**

- Could you give me a brief overview of how your injury occurred and how we got to this point?
- Thinking back to your initial injury, when did you first notice that something wasn’t right?
- What were your main concerns? (Why did X concern you?)
- How did healthcare professionals respond to you and your injury? (How did X make you feel?)
- What did treatment initially consist of? (How did you feel about X?)

**Initial suggestion of CRPS**

- How long did it take for you to be told you had CRPS? (What do you think about that?)
- How did you feel when you were told you had CRPS? (Why X?)
- Where did you get most of your info about CRPS/who did you discuss this with? (How did this make you feel?)
- Were there any differences in information that you were receiving about CRPS, including from what you read? (What were these / What do you make of that?)
- How would you explain what CRPS is to a friend?

**Treatment for CRPS**

- How did treatment for your injury change once CRPS was suspected/confirmed? (What did you think about X?)
- What do you understand the purpose of X treatment to be?
- At this point, what area of your life was CRPS affecting the most? (Why was this important?)
- What were your priorities from treatment? (Why X?)
- How did you feel healthcare treatment addressed your priorities/who you are as a whole? (Why X?)
- How much of your treatment do you do at home by yourself? (What is this like / how do you feel about this?)
- How long do you think that your CRPS will continue? (Why?)
- How do you know that you are getting better?
- How would you decide when to stop treatment?

**Summary**

- Where are you at in your CRPS journey right now?
- What is your sign/symptom that affects you the most currently? (What is the effect of this?)
- If you could influence one thing about future healthcare for people with CRPS, what would it be?
- Before we conclude this interview, is there anything about your experience of healthcare that we haven’t had a chance to discuss?

---------------------------------------------------------------------------------------------------------------------

**Numbers for support:**

[1737, Need to talk?](https://www.1737.org.nz/) Free call or text 1737 any time for support from a trained counsellor

[Lifeline](https://www.lifeline.org.nz/) – 0800 543 354 or free text HELP (4357)

[Samaritans](http://samaritans.org.nz/) – 0800 726 666

[Suicide Crisis Helpline](https://www.lifeline.org.nz/suicide-crisis-helpline) – 0508 828 865 (0508 TAUTOKO)
