# Supplementary material for: Experiences of diagnosis and treatment for upper limb Complex Regional Pain Syndrome: a qualitative analysis
Source: Pain Med. 2023 Aug 16;24(12):1355–63. doi: 10.1093/pm/pnad111 (PMC10690851; doi:10.1093/pm/pnad111)
Supplement: pnad111_Supplementary_Data [file pnad111_supplementary_data.zip › pnad111_Supplementary_Data/Appendix B Themes, sub-themes, and codes.docx]

**Appendix B: Themes, sub-themes, and codes**

| **Themes** | **Sub-themes** | **Codes** |
| --- | --- | --- |
| Wanting to get back to the person I was | - *An injury to the hand impacts everything* | Cost me more than the use of my hand  Social life has cut back  Quite tough mentally |
|  | - *Hit by one thing after the other* | I’ve had other things crop up  Medication side effects |
| Don’t know what’s going on | - *Something’s not right* | Signs and symptoms  A bigger problem than communicated |
|  | - *Kept in the dark* | I just needed more information  Involve me in the process |
| Not taken seriously | - *There’s a person in here* | Dismissed  You get that feeling  Rushing  Maybe I didn’t communicate  Space for listening  Follow up  Understand the impact  Nurturing |
|  | - *Your scans show no reason* | All in your head  The way you say it |
| Another layer of load | - *No-one wants to diagnose me* | CRPS is hard to diagnose  Reluctant to diagnose  Clinician skills/knowledge/experience  Conflicting opinions  Timing of diagnosis  I think you might have…  Get to it really quick |
|  | - *Battling with bureaucracy* | Accusatory  Navigating the system |
|  | - *All the different appointments and things* | Can I afford to go  Waste of time  Hard to remember  Waiting  I would repeat and repeat and repeat  A coordinated response |
| Trying not to let it stop me | - *Working around it* | Adapting  Changing attitudes  Strategies  No longer trapped |
|  | - *Doing everything suggested* | Pharmacological treatments  Non-pharmacological treatments:  Desensitisation  Retrain your brain  Cognitive approaches  Support for your hand  Sensory things  Things that work for me |
|  | - *Noticing the little milestones* | Seeing progress  Bigger picture  Changing priorities  Normal life  Visual changes  Pain reduction |
